# Machine learning for stone artifact identification: Distinguishing worked stone artifacts from natural clasts using deep neural networks

**DOI:** 10.1371/journal.pone.0271582

**Published:** 2022-08-10

**Authors:** Joshua Emmitt, Sina Masoud-Ansari, Rebecca Phillipps, Stacey Middleton, Jennifer Graydon, Simon Holdaway

**Affiliations:** 1 School of Social Sciences, University of Auckland, Auckland, New Zealand; 2 Centre for eResearch, University of Auckland, Auckland, New Zealand; 3 Office of Research Strategy and Integrity, University of Auckland, Auckland, New Zealand; Vellore Institute of Technology: VIT University, INDIA

## Abstract

Stone artifacts are often the most abundant class of objects found in archaeological sites but their consistent identification is limited by the number of experienced analysts available. We report a machine learning based technology for stone artifact identification as part of a solution to the lack of such experts directed at distinguishing worked stone objects from naturally occurring lithic clasts. Three case study locations from Egypt, Australia, and New Zealand provide a data set of 6769 2D images, 3868 flaked artifact and 2901 rock images used to train and test a machine learning model based on an openly available PyTorch implementation of Faster R-CNN ResNet 50. Results indicate 100% agreement between the model and original human derived classifications, a better performance than the results achieved independently by two human analysts who reassessed the 2D images available to the machine learning model. Machine learning neural networks provide the potential to consistently assess the composition of large archaeological assemblages composed of objects modified in a variety of ways.

## Introduction

The ability to distinguish natural from human manufactured stone artifacts has a long history in archaeology. It formed the basis for resolving the eolith controversy last century [1] and it continues to feature in discussions around the veracity of claims for pre-terminal Pleistocene human occupation in South America [2]. It also features in the identification of very early African stone artifacts, for example those from Lomekwi 3 [3,4]. Most recently, the situation has become more complicated with the well documented creation of unintentionally flaked stone artifacts by wild bearded capuchins in Brazil, suggesting that the definition of what constitutes “naturally flaked” objects needs careful consideration [5–7]. Which types of organisms created flaked stone artifacts and what types of behaviors they imply is now the topic of much research [8], as is the use of rock clasts in a variety of forms that were not flaked [9]. Indeed, there are suggestions that intentionality need not account for the manufacture of a number of recognized artifact forms created by hominins [10].

Despite this flurry of activity, methods intended to differentiate unmodified from modified objects have not developed significantly since those identified in the twentieth century. A variety of attribute based approaches are described but despite these there is a continued reliance on the opinion of experts who are able to identify the attributes of conchoidal fracture, and therefore make determinations about the anthropogenic origin of flaked objects (or indeed in the case of the Capuchins, an anthropoid origin) (e.g., [5]). This raises issues around access to such experts and of course consistency in the identifications. Another set of issues concern the volume of material that needs to be identified. In many instances gravel sized rock clasts including those potentially showing anthropogenic or anthropoid modification are abundant, so separating classes of modified from unmodified material may involve large numbers of individual identifications. Such decisions are important not only for identifying the extent of past activity but indeed for determining whether rock fracture is likely to reflect anthropogenic activity or not, since one criterion discussed in the literature on early occupation sites refers to the abundance and proportion of modified versus unmodified objects (e.g., [3,4]).

**Fig 1:**
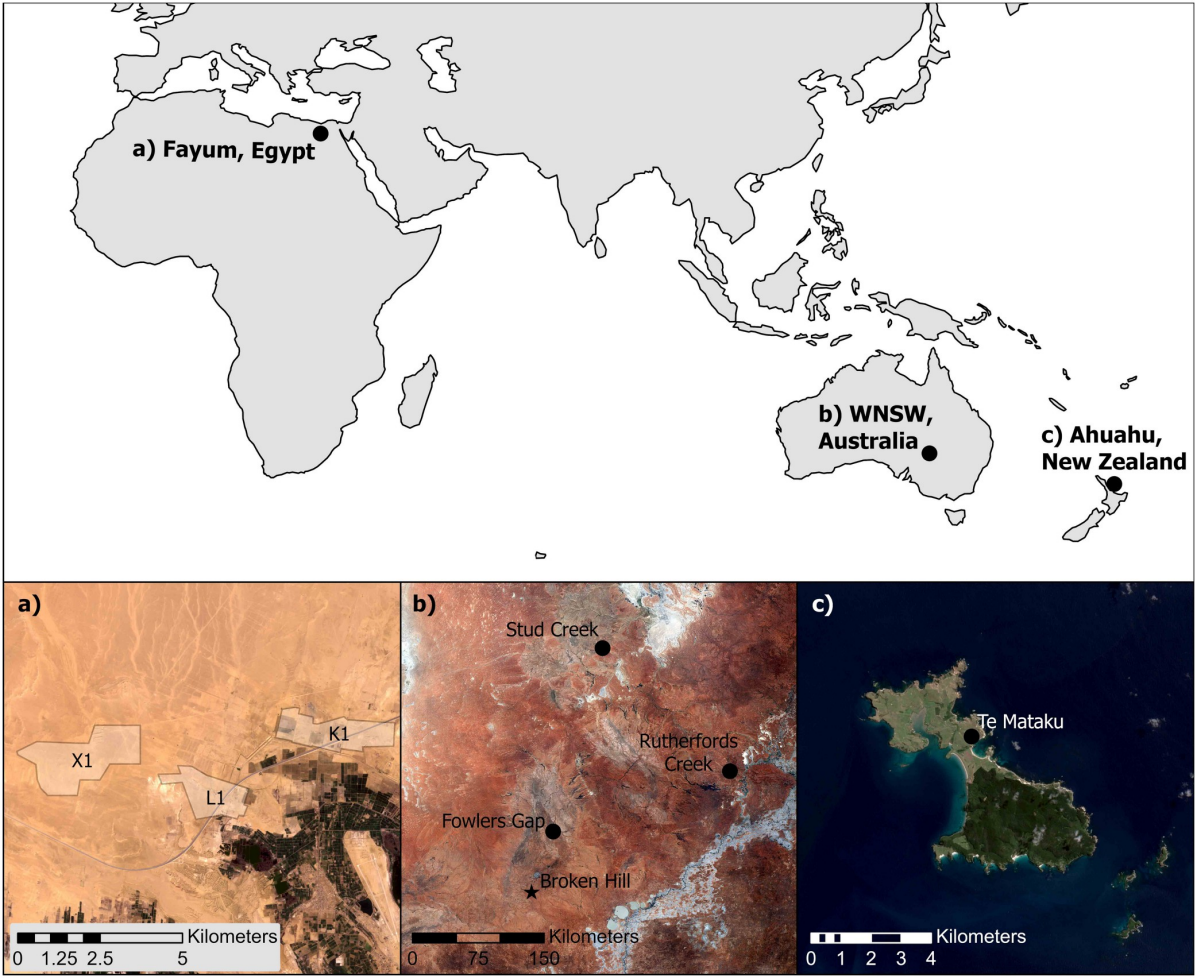
Locations of the three case studies. a. Fayum north shore showing the X1, L1, and K1 study areas [11,12]; b. western New South Wales Archaeological project areas with the town of Broken Hill shown for reference [13–15]; c. Te Mataku archaeological site Ahuahu, New Zealand [16]. Produced from ESA remote sensing data. Contains Copernicus Sentinel data [2022]. Satellite imagery from Sentinel-2 data available at https://scihub.copernicus.eu/; Country outlines from https://www.naturalearthdata.com/.

Our interest in stone artifacts and naturally occurring rocks relates to the issues that arise when confronted by their abundance in some archaeological sites versus the limited number of archaeologists with experience in their identification, a particular problem in some commercial archaeological resource management projects. Here we report work intended to create a machine learning based technology for stone artifact identification as part of a solution to the lack of experts available to distinguish large quantities of worked stone objects from naturally occurring clasts. In developing this technology we address two sets of questions: first, what do we mean when we talk about categories of modified and unmodified stone objects, and second, how does machine learning provide a solution to the task of classifying stone objects? The data sets we use come from three separate regions, stone artifact records from surface deposits in the Fayum region of Egypt, similar records obtained from western New South Wales, Australia, and stone artifact records excavated from Ahuahu, a small offshore island in New Zealand (Fig 1). Although these regions are geographically and culturally distinct, stone artifact identification in all three used similar recording protocols. In all three projects data recording also involved taking two dimensional photographs for artifacts providing over 3868 stone images for machine learning model development and testing.

Machine learning for object identification finds a diverse range of applications, from robotic vision and autonomous driving to security systems [17]. Scientific examples include applications like the automatic identification of species in ecological studies (e.g., [18–20]). This increase in applications is due both to the availability and accuracy of machine learning algorithms such as YOLO [21], R-CNN [22], and Faster R-CNN [23]. Two-stage detectors first identify regions of interest in an image and then classify them, greatly increasing the accuracy of objects identified [17]. Machine learning implemented in archaeological research most often involves the identification of features from remote sensing data (e.g., [24–26]) but while its application to object identification is less common it has proved useful for the classification of objects within typological sequences (e.g., [27]). Here we build on previous applications of machine learning in archaeology and explore its use for rapidly and accurately identifying large numbers of fractured and non-fractured rocks.

## Differentiating modified from natural stone objects

People in the past flaked stone to form artifacts using percussive force initiating conchoidal fracture and through this creating modified objects with distinctive flake scars, the morphology of which are well understood [28]. Most expert stone artifact analysts focus on the production of stone artifacts, recording attributes associated with conchoidal fracture and different flaking strategies. However, if the goal is to understand human behavior in all forms, not simply that related to the production of specific artifact types, then consideration is needed of the widest range of possible artifacts. Conchoidal fracture often indicates intentional manufacture, but not always, as there are circumstances when environmental processes will produce such fractures. Studies conducted in different parts of the world have proposed artifact attribution based on the frequency and proportion of technological attributes of conchoidal fracture, as well as a consideration of the contexts in which objects were subject to modification (e.g., [29–33]). However, conchoidal fracture initiated via percussion is of course not the only means by which rocks are modified. Fracture also occurs through actions like trampling reflecting a different form of human activity than manufacture [8,34–36]. The composition of artifact assemblages created through percussive initiated conchoidal fracture and rock clasts subject to trampling will be different, but identifying one group as artifactual and the other as natural relates to the assumed intentionality of the modification rather than the ability to draw inferences about behavior more generally.

Heat fracture of rocks produces another class of modification [37]. Rocks were used as heat retainers connected with particular pyro technologies developed in many places and times in the past. Concentrations of heat retainers found frequently in archaeological contexts indicate the locations of hearths or earth ovens. However, fire cracked rocks are also found distributed across some archaeological sites not associated with fire features. In many cases, fire cracked rocks show heat induced fractures but at times flaked and ground stone artifacts were also used as heat retainers. These objects are sometimes associated with fire features, but also might occur in more dispersed scatters. There are also examples of heat modification to facilitate flaking [38]. As with the products of trampling, fractures from heating may indicate human activities suggesting that fire cracked rocks be considered as artifacts (although heat fracture may also occur without human activity in a variety of situations).

Rocks were also modified in ways that did not leave flake scars. Rocks might be moved from one location to another for example, with the most visible examples involving those with a geological origin foreign to the location in which they are found [39]. Such rocks might be identified as artifacts if concentrated through human action (or indeed the actions of non-human primates).

In sum, stone artifacts preserve in many contexts and following the ‘man the tool maker’ trope, it is understandable why the distinction between flaked stone artifacts and unmodified rocks has drawn attention. However, quite apart from the removal of the gender prescription, archaeologists now recognize a much broader range of behaviors associated with stone artifacts including those discussed above. This raises questions about the range of materials to be recorded during archaeological investigations consistent with ideas about what might constitute modified stone artifacts following from a broader understanding of intentional human behavior. It remains important to identify flaked stone artifacts but it may also be important to understand levels of fragmentation in both flaked and non-flaked rock clasts, including clasts that are heat fractured. It may also be necessary to compare the quantities of such modified stone objects with the quantities of rock clasts that are not modified in these ways. This obviously increases the number of objects that must be observed and categorized, with these quantities potentially reaching into the tens to hundreds of thousands of objects.

At issue here is not only the time that such identification might take but also the expertise to undertake such identification. Consistent identification requires skill, and even if basic knowledge is acquired identification may be difficult due to raw material type, or the fragmentation of the object. Most practitioners, even the most inexperienced are able to record some metric information, but the ability to classify artifacts is more challenging. Moreover, there is also the question of agreement among such experts on how objects should be classified and how the certainty of object identification is measured.

If archaeologists are interested in understanding the processes that led to the accumulation of rock clasts of different types in archaeological sites then methods are needed that extend those currently available for stone artifact identification. One approach is to automate object identification through the application of machine learning technologies.

## Materials

Two dimensional photographs of stone artifacts recorded in the three case study archaeological projects from Egypt, Australia, and New Zealand provided a large data set with which to train and test the machine learning model described below. In the following we briefly describe the case study areas before providing details of the photographs.

*Case study 1*, *Egypt*: Photographs come from artifact data recorded during the URU Fayum project, a collaboration between the University of California Los Angeles, the Rijksuniversiteit Groningen, and the University of Auckland. Permission to work in the Fayum, Egypt was granted by the Supreme Council of Antiquities (now the Ministry of Tourism and Antiquities). A large scale surface survey was undertaken across the northern shoreline of Lake Qarun, Fayum, Egypt between 2008 and 2012 [12] recording surface scatters of flaked stone artifacts, almost exclusively flint [40]. A quality assurance protocol included photographing a random sample of artifacts with duplicate attributes recorded by independent analysts. All stone artifact attributes and measurements followed definitions in Holdaway and Stern [41] supplemented by information relating to North African tool typology. A small sample of un-modified cobbles from the Fayum (n = 36) were also photographed and recorded.

*Case study 2*, *Australia*: Data recording as part of the Western New South Wales Archaeological Program (WNSWAP) involving Macquarie University, the Australian National University, and the University of Auckland also involved a quality assurance protocol involving photographs of a random sample of artifacts and their double recording by an independent observer [42]. The stone artifact attributes and measurements taken also used the definitions provided in Holdaway and Stern [41]. The data records and photographs used in this study come from three WNSWAP projects: Stud Creek [13], Fowlers Gap [14], and Rutherfords Creek [43]. Silcrete and quartz are the most common raw materials used for artifact manufacture. Data collection occurred during the 1990s and into the 2000s with permissions provided by the Broken Hill Local Aboriginal Land Council, New South Wales National Parks and Wildlife Service, and the University of New South Wales Fowlers Gap Arid Zone Research Station Management Committee.

*Case study 3*, *New Zealand*: Data from the New Zealand case study comes from the analysis of material from the site of Te Makatu, a site excavated as part of The Ahuahu Great Mercury Island Archaeological Project, a collaboration between the University of Auckland, landowners, and tangata whenua Ngati Hei ki Wharekaho (the Māori tribe with ancestral connection and authority) [16,44–48]. In addition to flaked stone artifacts, a sample of rocks were also excavated. Permits to conduct the archaeological work on Ahuahu were obtained from Heritage New Zealand.

The photographs of artifacts and rocks from Egypt and Australia are from legacy data derived from completed projects, whereas those from the New Zealand case study are from an ongoing project with access to photographs as well as the physical objects. The three projects ran sequentially with the Australian project beginning in the 1990s, the Egyptian project beginning in 2008, and the New Zealand project in 2012. As a consequence, camera technology changed considerably. At Stud Creek, Australia photographs from the 1990s were taken on a Kodak digital camera producing a kdc file format. Those at Fowlers Gap used a Nikon Coolpix 900 (E900S) compact digital camera producing jpg files while those at Rutherfords Creek used Canon SLR digital cameras also producing jpg files. Canon cameras were also used in the Egyptian and New Zealand projects producing jpg files.

Since photographs were taken in the field in Egypt and Australia, backgrounds included natural surfaces (e.g., sand). This potentially influenced the identification of objects versus rocks since most of the later examples came from the New Zealand case study and were photographed in laboratory conditions with white backgrounds, as was the entire New Zealand flaked artifact sample. This difference in backgrounds raises the issue of model overfitting, where features specific to the dataset are learnt instead of features that generalize well to unseen examples. In this study for example, the model could have associated the white backgrounds used in laboratory conditions as a strong indicator of rocks instead of surface patterns indicative of worked material. This would reduce instances of misclassification simply based on the background present in the image. To mitigate against this unwanted bias, 1442 photos of rocks from New Zealand were photographed on a tray of sand as a means to reduce the influence of image backgrounds on feature learning.

Different raw material types have different flaking properties leading to variability in the visibility of the features of conchoidal fracture that archaeologists use to identify a stone artifact and therefore also machine learning classifications. For example, a siliceous, fine grained raw material (e.g., flint) may exhibit clearer landmark features of conchoidal fraction than a coarse grained material (e.g., silcrete). Table 1 summarizes the frequency of artifacts manufactured from different raw materials used as the training set and test for the machine learning model (described below). While the majority are fine grained (chert and flint), just under 800 examples are coarser grained materials, quartzite and silcrete helping to mitigate the influence of raw material types.

**Table 1:** Frequency of the stone artifact and rock raw material types used to train the machine learning data model, by case study location.

	Basalt	Bottle glass	Chert	Flint	Limestone	Petrified Wood	Pumice	Quartz	Quartzite	Rhyolite	Sandstone	Scoria	Silcrete	Total
***Stone Artifacts***														
New Zealand			2033											**2036**
Australia		2				3		283	76		2		696	**1067**
Egypt				771		2								**777**
Total		2	2033	771		5		283	76		2		696	**3868**
***Rocks***														
New Zealand	2342		57		53	8	148			197	28	33		**2866**
Egypt				35										**35**
Total	2342		57	35	53	8	148			197	28	33		**2901**

## Methods and results

Stone artifacts have some advantages when seeking classification by either humans or machines. The forms that manufactured artifacts take are constrained by the processes that lead to fracture. This means that supervised learning based on prior classifications is possible [49]. For example, using the approach advocated by Peacock [33], a human observer might assess a series of artifact attributes. Peacock suggests such things as bulbs of percussion, radial lines and bulbar scars, the number and concentration of these scars, and their orientation in relation to the axis of the object as diagnostic attributes (cf. [41]). These attributes, Peacock argues, are more reliable than a focus on the overall shape of the object when assessing whether an object is manufactured or not.

In our study, initial identification and recording of manufactured stone artifacts used a supervised classification following Peacock’s advice. Initial identification occurred in three separate field contexts (Materials) with artifact attributes recorded by analysts and photographed. These photographs and associated artifact classification form both the training and testing sets in the current machine learning experiment. In a separate, but related exercise, otherwise unmodified rocks were selected from collections held at the University of Auckland Archaeological Laboratories and similarly assessed and photographed.

**Fig 2:**
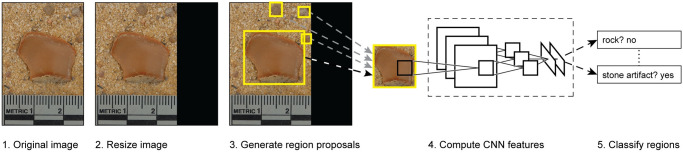
Example of a convolution neural network (CNN) workflow.

Machine learning as applied here works in a different way to the attribute based classification of rocks versus flaked artifacts proposed by scholars like Peacock [33]. Based on the results of a training set of human classifications, machine learning analyzes images refining this classification to match classification categories. The Sobel filter provides an illustration of how this might be achieved. In image analysis, the filter uses a 3x3 matrix of numbers (called a kernel) and applies this to each pixel in an image (convolved with the image). The result is a new image that emphasizes edges. In machine learning, a convolution neural network does the same thing, except that it learns the kernel itself and can learn and apply large numbers of such filters (Fig 2). When a filter is applied to an image, it results in a ’feature map’, basically a heatmap of where that feature is present. A neural network learns many of these in parallel. The feature map is then treated as another image, creating what might be thought of as a hierarchy of feature maps and filters. At the lowest level in this hierarchy, feature maps reflect things like small inclusions in the rock, while at the highest level the feature maps learn things like artifact and rock shapes, and includes the edges within these shapes. The name, deep neural network, refers to the existence of a chain of kernels and feature maps. However, the successive images in the filters do not represent attribute ‘features’ as Peacock [33] describes but are better thought of as a series of increasingly refined object abstractions.

In our application of an object detection workflow based on deep neural networks to locate and classify artifacts and rocks in images, the dataset consisted of 6769 images, 3868 artifacts and 2901 rocks. Each image contained only one object of interest. Bounding boxes were defined by human analysts viewing each image and drawing a box around the maximum extent of the artifact in the image using the KITTI software [50]. Images and their annotations were scaled to 300 x 300 pixels to keep processing requirements low. Aspect ratios were maintained, and black pixels were used to fill the remaining space.

An openly available PyTorch implementation of Faster R-CNN ResNet 50 was used as the object detection model architecture [51,52]. Faster R-CNN [23] is a region proposal network that learns to identify regions of interest in an image as well as classify them using a set of shared convolutional neural network features. The convolutional neural network (CNN) architecture has become the state-of-the-art in image processing methods [53]. The ResNet component of the model is an implementation of residual networks [54], a method of improving CNN performance by allowing for much deeper, and thus more complex neural networks to be trained effectively.

A set of 965 images were set aside as a benchmark to evaluate human and model performance for single object detection. After training, the resulting deep neural network implementation was evaluated against the benchmark set of images to determine the accuracy of differentiation compared to the original human classification (obtained either in the field for the Egyptian and Australian case studies, or in the laboratory for the New Zealand case study). A second evaluation asked two human analysts to reclassify the benchmark set of images without access to the original classification (or the actual objects). Below the results of these two classification exercises are compared. The benchmark set of images consisted of 268 rocks, 278 flakes, 249 tools, and 170 cores. This implementation of machine learning did not seek to differentiate flakes, cores, and tools, but the inclusion of objects from all three categories ensured variation in size and shape within the manufactured artifact category. For model evaluation, the benchmark images underwent the same pre-processing described above (i.e., bound-box creation and scaling). However, for the human benchmark set evaluation a different process was followed where the bounding box of the object was used to crop it from its background at the original resolution leaving only images for the analysts to examine limited to the same KITTI bounding boxes at 300 x 300 pixel resolution available to the machine. This removed any advantage a human participant might have in classifying artifacts based on their relative size as well as providing a measure of the upper bound of human accuracy on a full resolution image. This change is important because in effect it removed the availability of contextual variable clues for the human participants. Whereas in the original field classification where the analyst was able to hold and manipulate the stone artifact while recording attributes, we now asked the analyst to classify the object based on a 2D image alone, in other words using the same data form assessed in the machine learning experiment.

**Fig 3:**
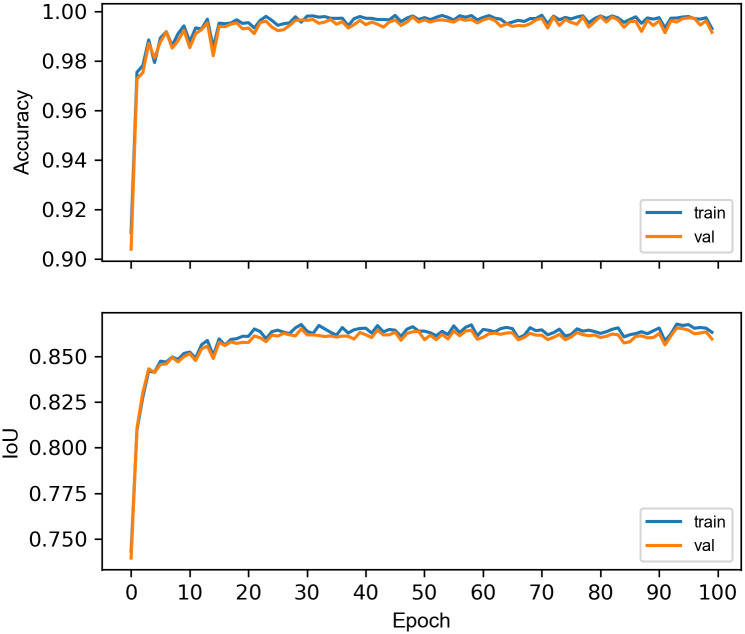
Training (train) and validation (val) mean accuracy and IoU scores for 10 fold cross-validation over the set of 5804 training images. Results suggest minimal overfitting to the training data however additional improvements to accuracy and IoU were limited after 30 epochs.

In our experiment, the machine learning model was trained on the remaining 5804 annotated images (manufactured stone artifacts and rocks) using the Adam optimizer [55] with a learning rate of 0.001 and a batch size of 16. To prevent exploding gradients, these were clipped to 1. Training was conducted for 30 epochs using image augmentation via vertical and horizontal flipping with probability set to 0.5 each. The number of training epochs were determined via 10 fold cross-validation over 100 epochs with validation after every epoch (Fig 3). The final model was trained on all available training data and took 6.8 hours on an NVIDIA P40 GPU.

The trained model generated multiple region proposals for an image, each with a classification output score between 0 (low) and 1 (high) for each class label. For evaluation purposes, the proposals were ranked by their associated classification scores, with the classification label associated with the highest score used as the predicted class for the image. Since each image only contained one object, this was considered sufficient to determine the object’s classification.

The machine learning model correctly classified 100% of the benchmark set compared to the original in field or in laboratory classification, with a mean intersection over union (IoU) score of 0.82 and a standard deviation of 0.13 for the predicted bounding box. This ratio compares the accuracy with which the model was able to identify the bounding box added manually to the classification images. Ratio values greater than 0.70 are considered good. The two human participants who independently reclassified the benchmark set achieved an accuracy of 99.4%, and 94.8% respectively, that is close to but in both cases slightly lower than the machine learning model. Based on these results, using 2D images, it is possible to consistently classify conchoidally flaked stone artifacts from rocks across a range of lithologies, where stone artifacts are sourced from archaeological assemblages deposited in different places and time periods.

## Discussion

This initial application used a machine learning model to distinguish between only two classes of rock clast, those with evidence of conchoidal fracture and those without. The machine learning algorithm determined the classification based on previous human identifications and did not take the context of the objects into account. This means the machine learning algorithm on its own could not definitively tell us that an object was an artifact. Unworked objects from the three test cases might still be artifactual in the sense that they were accumulated by human or non-human primates, and conchiodal fracture may have occurred under natural circumstances. However, the machine learning experiment does overcome the issue that Peacock [33] raised about the use of experts. In effect, machine learning model iterated human expertise by modelling this expertise and allowing the result to be applied in multiple contexts, including those where the original human experts were not present. Moreover, the model applied this expertise in a uniform manner to large numbers of objects. With the need for the physical presence of experts removed, and the ability to duplicate the instantiations of the software, it becomes possible to analyze very large assemblages rapidly in a variety of settings including for example, museums.

The initial classifications of the objects used in this experiment were created by humans who examined the physical objects. This means that the whole object could be considered, as opposed to a single side of an object as is the case with a 2D photographic image. The results of the two human participant identifiers assessed against the machine learning model suggested that the model was more accurate. However, the way objects were identified differed between humans and the model across the stages of the experiment. Humans initially considered the objects as a whole in the field or in the laboratory and classified these based on their previous experience and observations, including an understanding of the context in which they were found. The machine learning model used the results of these observations to develop an image based classification scheme. Therefore, it also used previous experience but without the aid of context and size. Subsequently, human participants were tested against the machine but only by giving them access to the 2D photographs, with context and size removed, which explains their inability to better the machine learning model. The advantage machine learning provides compared to the human analysts is that its identifications were learnt and determined statistically, whereas those by humans potentially incorporated elements of doubt or second-guesses. Essentially the model is more consistently objective than the humans.

The obvious next step is to develop the training sets further with the goal of increasing the machine learning classification beyond the two classes identified in the current work. However, this involves not only additional training sets but also improvements to object images, moving to 3D images that provide the complexity needed to classify fractured objects from conchoidal cores to fire fractured rock. Improvements are also needed in the software to enable more complex machine learning model development. With three dimensional models comes the ability to not only classify objects, but to derive data on size and geometric measures of shape.

The 2D images from the Egyptian and Australian case studies were taken in the field. The human analysts used in the machine learning experiment noticed variability in terms of image quality, with some images out of focus making the objects hard to identify. Differences in image quality illustrate another advantage of the machine learning model. Where humans look for platforms, dorsal scars, and bulbs of percussion (amongst other features, following Peacock [33]), the model builds an image based classification system. This may be extremely useful when dealing with raw materials like quartz where the features associated with conchoidal fracture are not obvious. It also has potential for dealing with already extant photographic collections where the quality of the images may be variable.

Finally, there are several additional avenues for future research with this methodology. In the case of stone artifacts the aim would be to expand the analysis to the classification of types of stone artifacts such as flakes, cores, and tools, and differences therein. One of the challenges in doing this is not just to identify such objects but to identify when a stone artifact may meet the criteria for several classification types and therefore not belong to a single class, following for example, the reuse of artefacts previously discarded. Artifact classification is not straightforward as many scholars have pointed out, with objects manufactured, used, and modified many times over prior to discard changing their function (e.g. [56,57]). The ability to identify features that relate to these changes would permit life histories of the objects to be recorded.

## Conclusions

Using stone artifact images recorded from archaeological sites in Egypt, Australia, and New Zealand, and a sample of unmodified rocks, a machine learning classification model was developed. The training data derived from object identifications undertaken in the field and in a laboratory setting together with 2D photographs. The machine learning model performed well when tested against two human analysts given access to the same 2D photographic data sets as the machine. Machine learning is a promising technology for classifying large numbers of rock clasts present in archaeological sites and helping to determine their behavioral significance. While the artifactual status of objects depends on context as well as object form, machine learning models provide the ability to rapidly and objectively classify objects based on previous identifications. Developments in this technology and future applications offer the potential to greatly enhance the ability to analyze all the rock casts that make up archaeological assemblages.

## Supporting information

S1 File (DOCX)
