# Supplementary material for: Machine learning for stone artifact identification: Distinguishing worked stone artifacts from natural clasts using deep neural networks
Source: PLoS One. 2022 Aug 10;17(8):e0271582. doi: 10.1371/journal.pone.0271582 (PMC9365149; doi:10.1371/journal.pone.0271582)
Supplement: S1 File — (DOCX) [file pone.0271582.s001.docx]

Inclusivity in global research

PLOS’ policy on inclusivity in global research aims to improve transparency in the reporting of research performed outside of researchers’ own country or community and ensures that PLOS publications reporting global research adhere to high standards for research ethics and authorship. Authors of relevant research articles may be asked to complete the questionnaire below, which outlines ethical, cultural, and scientific considerations specific to inclusivity in global research. This questionnaire may be requested when researchers have travelled to a different country to conduct research, if research uses samples collected in another country, research with Indigenous populations or their lands, or if research is on cultural artefacts. Researchers travelling to another country solely to use laboratory equipment will not normally be required to complete the questionnaire. However, the questionnaire can be requested at the journal’s discretion for any submission – if you have been requested to complete this questionnaire by the PLOS journal you submitted to, please do so.

Please complete the questionnaire below and include this as a Supporting Information file with your manuscript. Note that if your paper is accepted for publication, this checklist will be published with your article in the supporting information files. Please ensure that you reference the checklist in the main body of your manuscript. We suggest adding a subsection ‘Inclusivity in global research’ to your Methods section and adding the following sentence: “Additional information regarding the ethical, cultural, and scientific considerations specific to inclusivity in global research is included in the Supporting Information (SX Checklist)”

The questions have been designed to be applicable to a wide range of study types, and there are subsections for both human subjects research and non-human subjects research. If any of the questions are not relevant to your research please mark them as “N/A” as appropriate.

**Ethical considerations, permits and authorship**

*This section is applicable to all research types.*

Provide details as to who granted permissions and/or consent for the study to take place in the Methods section of your manuscript. This should include the names of **all** ethics boards, governmental organizations, community leaders or other bodies that provided approval for the study. If individuals provided approval refer to these people by their role or title but do not list their name(s).

Reported on page number: 10

If there were any deviations from the study protocol after approval was obtained please provide details of these changes in the Methods section of your manuscript.
Did this study involve local collaborators that are residents of the country where the research was conducted or members of the community studied? If you do not have any authors from said communities, please provide an explanation for this below.

Reported on page number: N/A

No. In the cases of the Australian and Egyptian data the data was collected 10 years or more previously with permission of indigenous communities and appropriate government agencies. As these field projects are no longer active, participants are no longer in contact. The Aotearoa data was collected with the knowledge and consent of relevant indigenous communities, but they did not have an active part in this analysis.

Everyone listed as an author should meet PLOS’ criteria for authorship and all individuals who meet these criteria should be included in the author byline, rather than the acknowledgements. Authorship criteria is based on the International Committee of Medical Journal Editors (ICMJE) Uniform Requirements for Manuscripts Submitted to Biomedical Journals - for further information please see here: <https://journals.plos.org/plosone/s/authorship>.

**Human subjects research (e.g. health research, medical research, cross-cultural psychology)**

Did you obtain written informed consent from a representative of the local community or region before the research took place? How did you establish who speaks for the community? Details of written informed consent obtained from study participants should be reported separately in the Methods section of your manuscript.

N/A

How did members of the local community provide input on the aims of the research investigation, its methodology, and its anticipated outcome(s)?

N/A

When engaging with the local community, how did you ensure that the informed consent documents and other materials could be understood by local stakeholders?

N/A

Will the findings of the research be made available in an understandable format to stakeholders in the community where the study was conducted (e.g. via a presentation, summary report, copies of publications, etc.)? Please provide details of how this will be achieved.

N/A

**Non-human subjects research using specimens/ animals collected as part of the study, or those housed in archival collections. Examples include archaeology, paleontology, botany and zoology.**

Did the permission you obtained from a local authority to perform the study include an agreement on access to outputs and benefit sharing? This may include procedures to enable fair distribution of the benefits and resources arising from the research performed. Please include any details of Prior Informed Consent and Benefit Sharing Agreements obtained. These may be required by field-specific regulations, for example the Convention on Biological Diversity (CBD) and the associated Nagoya Protocol.

In all cases authorites to carry out archaeological research require preparation and disseminiation of reports on all research activities and we have done this in all three cases. While these types of agreements do not always specific academic outputs, we have also included academic publications and whereever possible these have been shared. Previous and current archaeological permissions do not include statements on digital data and archaeologists are expected to follow current best practice.

If the material used in your study was imported, please A) provide the year it was imported and B) indicate whether permits were obtained to import/export the materials used, C) provide details of any permits obtained. If this information is not available, please indicate this.

No material was imported

If you used archival specimens, please state how the material used in your study was acquired by the institute it is held in and provide details of any permits obtained for the original excavations/ sample collection. If this information is not available, please indicate this.

The images used by this study from Egypt and Australia were part of previous archaeological research projects as outlined below. They were collected in the countries of origin and are now stored digitally at the University of Auckland.

The Australian Western New South Wales Archaeological Program was directed by Patricia Fanning and Simon Holdaway. Permissons for the work were granted by the Barkinji people as the traditional owners of country around Rutherfords Creek, and Fowlers Gap, and the Wangkumara Cultural Heritage Management Committee, and the Tibooburra Local Aboriginal Land Council as traditional owners of the Stud Creek country. Images were taken between 1999 and 2007.

The Egyptian URU Fayum project received permission to work in the Fayum from the Egyptian Supreme Council of Antiquities and the Ministry of State for Antiquities (MSA) to the University of California Los Angeles, Groningen University, University of Auckland Fayum Project (URU). Images were taken between 2008 and 2012.

The Ahuahu Great Mercury Island Project received permission to work on Ahuahu under a authority granted by Heritage New Zealand, and in consultation with the tribal authority Ngāti Hei and with access provided by the landowners, the Fay and Richwhite families.

How was the potential cultural significance of the materials collected in your study to local communities considered in your research design? Were Indigenous peoples and/or local researchers and institutions involved with archaeological excavations / collection of specimens? If so, please provide a description of their involvement.

The WNSWAP project was groundbreaking in the sense that traditional owners at the outset of the project requested that no artefacts were removed from the landscape. This is particularly challenging for archaeologists, but the research team was able to develop an entire system of recording to ensure that not a single artefact was removed from the landscape. This approach was also employed in the Fayum, Egypt. Local communities were aware of the potential for development to impact the archaeological heritage of the region. Our approach to recording ensured comprehensive coverage of the landscape to document archaeological heritage that is now unfortunately destroyed. In the case of New Zealand regular meetings with the indigenous community and involving particpants in the fieldwork has ensured their priorities are part of our research design from the inception of the project. This includes incorporating their values and priorities into research, in addition to those of wider Māori communities in New Zealand. This is also partly a requirement of heritage legislation in New Zealand.

If your manuscript includes photographs of human remains please indicate whether authors obtained permission from descendants or affiliated cultural communities to do so.

N/A
